# Supplementary material for: On-Cell Saturation Transfer Difference NMR Spectroscopy on Ion Channels: Characterizing Negative Allosteric Modulator Binding Interactions of P2X7
Source: J Am Chem Soc. 2025 Aug 31;147(36):32400–11. doi: 10.1021/jacs.5c02985 (PMC12426935; doi:10.1021/jacs.5c02985)
Supplement: Supplementary file 1 [file ja5c02985_si_001.pdf]

On-cell Saturation Transfer Difference NMR spectroscopy on ion channels: characterizing negative allosteric modulator binding interactions of P2X7

Serena Monaco[1,2], Jacob Browne[1,3], Matthew Wallace[1], Jesús Angulo[4]*, Leanne Stokes[1]**

*Co-corresponding

[1] School of Chemistry, Pharmacy & Pharmacology, University of East Anglia, Norwich Research Park, Norwich NR4 7TJ, UK.

[2] Quadram Institute, Rosalind Franklin Road, Norwich Research Park, Norwich NR4 7UQ, UK.

[3] current address Department of Pharmacology, University of Cambridge, Tennis Court Road, Cambridge, CB2 1PD.

[4] Instituto de Investigaciones Químicas (IIQ), Consejo Superior de Investigaciones Científicas and Universidad de Sevilla, Avenida Américo Vespucio, 49, Sevilla 41092, Spain.

Corresponding author email addresses: Serena Monaco Serena.Monaco@quadram.ac.uk Jesus Angulo j.angulo@iiq.csic.es Leanne Stokes l.stokes@uea.ac.uk

Content

S1. Double difference STD NMR spectra for AZ10606120 with WT hP2X7.

S2. Double difference STD NMR spectra for JNJ-47965567 with WT hP2X7.

S3. Comparison of AZ10606120 and JNJ-47965567 position in the binding pocket from XRD of pdP2X7.

S4. Comparison of docking poses for AZ10606120 docked to WT or NAM-site mutant hP2X7.

S5. Comparison of docking poses for JNJ-47965567 docked to WT or NAM-site mutant hP2X7.

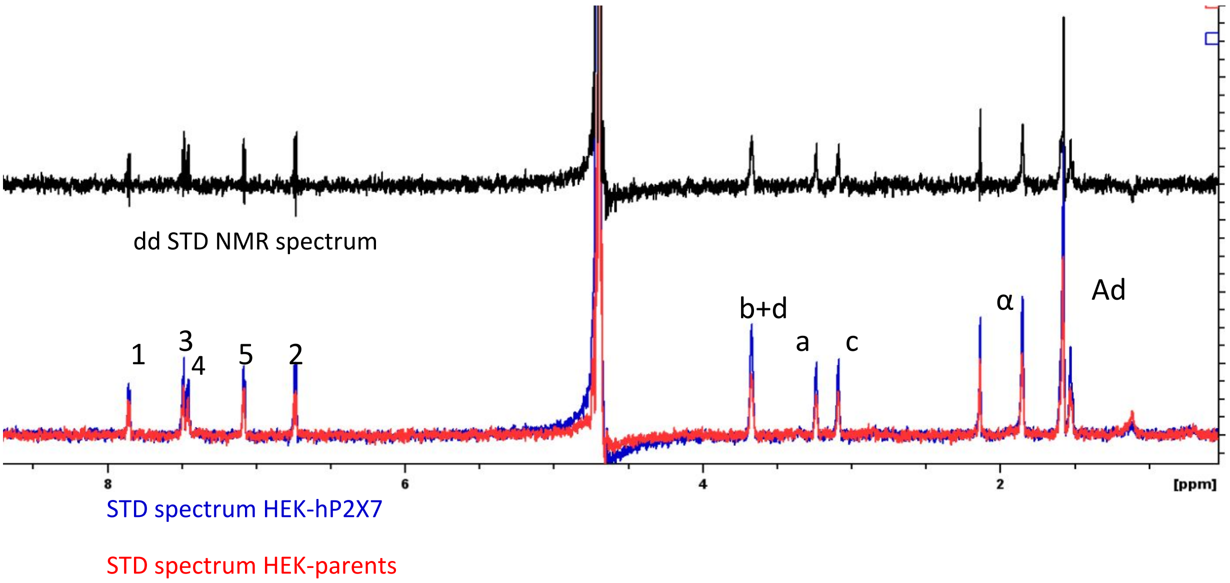

Supplementary Figure 1: double difference spectra for AZ10606120 bound to WT hP2X7.

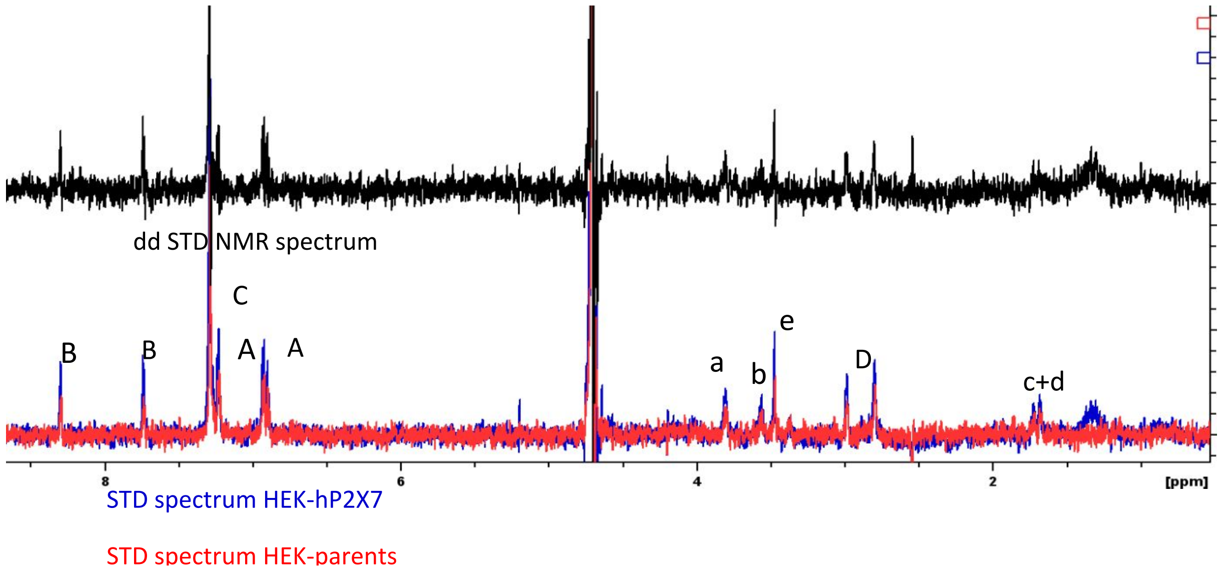

Supplementary Figure 2: double difference spectra for JNJ-47965567 bound to WT hP2X7.

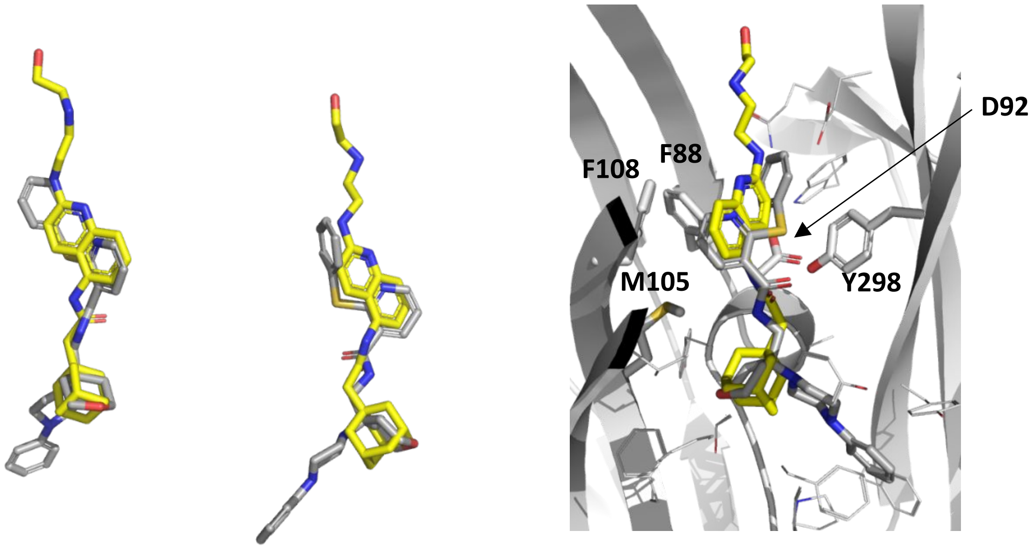

Supplementary Figure 3: Comparison of AZ10606120 (yellow) position in P2X7 NAM pocket with JNJ-47965567 (grey) position. Taken from crystal structure of pdP2X7 in complex with both ligands (pdb entries: 5u1w and 5u1x) [5].

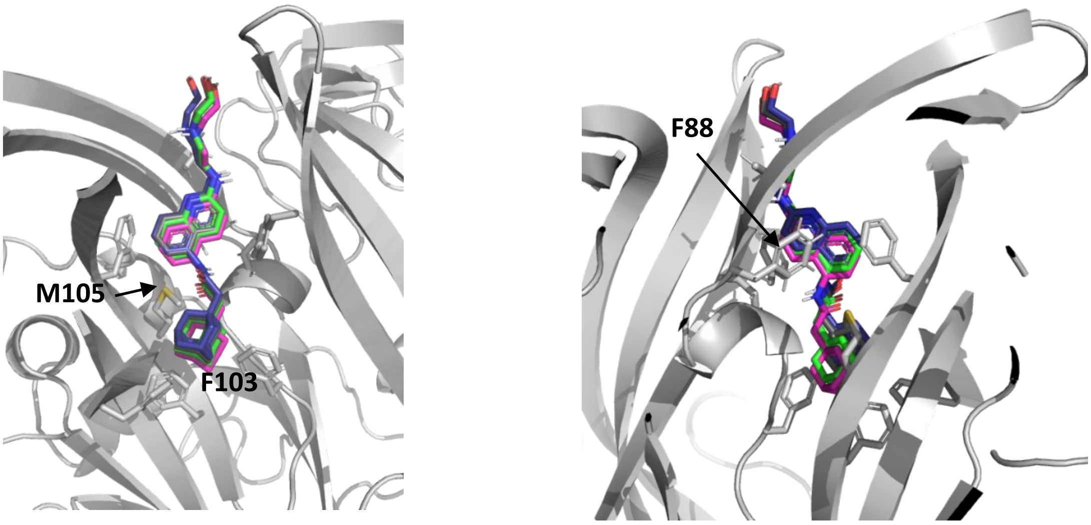

Supplementary Figure 4: Comparison of AZ10606120 docked to human P2X7 or NAM-site mutant hP2X7 with little variation in predicted pose. F88A-hP2X7 (blue), M105A-hP2X7 (green) and F103A-hP2X7 (magenta) poses shown overlaid with hP2X7 (grey).

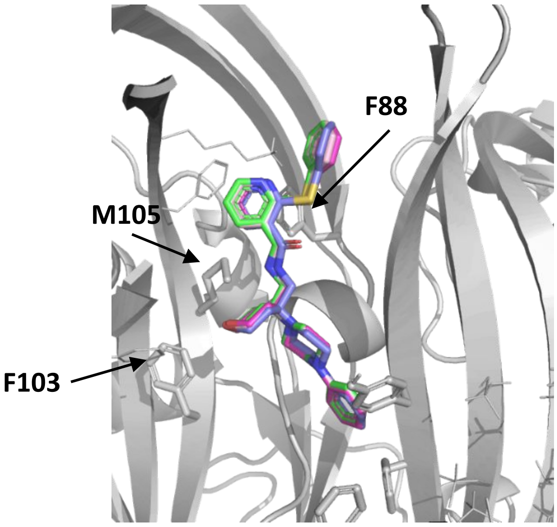

Supplementary Figure 5: Comparison of JNJ-47965567 docked to human P2X7 or NAM-site mutant hP2X7 with little variation in predicted pose. F88A-hP2X7 (blue), M105A-hP2X7 (green) and F103A-hP2X7 (magenta) poses shown overlaid with hP2X7 (grey).
